# Decorin induced by progesterone plays a crucial role in suppressing endometriosis

**DOI:** 10.1530/JOE-14-0393

**Published:** 2014-11

**Authors:** Yoshihiro Joshua Ono, Yoshito Terai, Akiko Tanabe, Atsushi Hayashi, Masami Hayashi, Yoshiki Yamashita, Satoru Kyo, Masahide Ohmichi

**Affiliations:** Department of Obstetrics and Gynecology, Osaka Medical College, 2-7 Daigaku-machi, Takatsuki, Osaka, 569-8686, Japan; 1 Department of Obstetrics and Gynecology, Graduate School of Medical Science, Kanazawa University, Kanazawa, Japan

**Keywords:** decorin, progesterone, dienogest, endometriosis, cell cycle arrest, p21

## Abstract

Dienogest, a synthetic progestin, has been shown to be effective against endometriosis, although it is still unclear as to how it affects the ectopic endometrial cells. Decorin has been shown to be a powerful endogenous tumor repressor acting in a paracrine fashion to limit tumor growth. Our objectives were to examine the direct effects of progesterone and dienogest on the *in vitro* proliferation of the human ectopic endometrial epithelial and stromal cell lines, and evaluate as to how decorin contributes to this effect. We also examined *DCN* mRNA expression in 50 endometriosis patients. The growth of both cell lines was inhibited in a dose-dependent manner by both decorin and dienogest. Using a chromatin immunoprecipitation assay, it was noted that progesterone and dienogest directly induced the binding of the decorin promoter in the EMOsis cc/TERT cells (immortalized human ovarian epithelial cells) and CRL-4003 cells (immortalized human endometrial stromal cells). Progesterone and dienogest also led to significant induced cell cycle arrest via decorin by promoting production of p21 in both cell lines in a dose-dependent manner. Decorin also suppressed the expression of MET in both cell lines. We confirmed that *DCN* mRNA expression in patients treated with dienogest was higher than that in the control group. In conclusion, decorin induced by dienogest appears to play a crucial role in suppressing endometriosis by exerting anti-proliferative effects and inducing cell cycle arrest via the production of p21 human ectopic endometrial cells and eutopic endometrial stromal cells.

## Introduction

Endometriosis is a chronic, benign disease, which is characterized by the presence of endometrium-like tissue outside the uterine cavity, primarily on the ovaries. It is a major cause of symptoms such as pelvic pain, dysmenorrhea, dyspareunia, and infertility. This condition affects 6–10% of females of reproductive age and often relapses after surgical therapy (Eskenazi & Warner 1997, Giudice & Kao 2004, Guo 2009). Gonadotropin-releasing hormone (GnRH) agonists are an established therapy for endometriosis. Although gonadotrophin-releasing hormone (GnRH) agonists provide effective pain relief and reduce the progression of endometriotic implants (Brown *et al*. 2010), they are associated with symptoms of estrogen deprivation, such as hot flushes, vaginal dryness, headache, and decreased libido. In addition, the involvement of GnRH agonists in bone demineralization restricts their use to 6 months in the absence of add-back therapy (Jee *et al*. 2009, Brown *et al*. 2010). Progestins are a class of compounds that mimic the activity of progesterone and are a recommended treatment for the pain associated with endometriosis, but a number of agents in this class are associated with androgenic effects and weight gain at doses that are required for efficacy (Vercellini *et al*. 2009).

Dienogest (17a-cyanomethyl-17b-hydroxy-estra-4,9-dien-3-one) is a fourth-generation progestin with a potent oral progestational activity without any systemic androgenic activity (Sitruk 2004*a*,*b*, Sasagawa *et al*. 2008). Results from clinical trials have indicated that dienogest provides effective pain relief equivalent to GnRH agonists, a reduction of endometriotic lesions, and a favorable safety and tolerability profile in patients with endometriosis (Cosson *et al*. 2002, Momoeda *et al*. 2009, Köhler *et al*. 2010, Strowitzki *et al*. 2010*a*,*b*, Felice *et al*. 2012). The effects of dienogest on endometriosis are associated not only with its anti-ovulatory activity (Irahara *et al*. 2007), but also with a direct effect on proliferation or cytokine production in stromal cells from eutopic and ectopic endometrial tissues (Okada *et al*. 2001, Horie *et al*. 2005, Fu *et al*. 2008). In a recent report, dienogest has been described as having a direct effect on endometrial epithelial cells (Shimizu *et al*. 2009). However, it is still unclear as to how dienogest affects endometriotic cells to contribute to its therapeutic effect on endometriosis.

Decorin, a prototype member of the small leucine-rich proteoglycan family (Kresse *et al*. 1993, Iozzo 1998, Neame & Kay 2000), is a stromal proteoglycan synthesized chiefly by fibroblasts, stressed vascular endothelial cells, and smooth muscle cells (Danielson *et al*. 1997, Keene *et al*. 2000, Zhang *et al*. 2006, Zhang *et al*. 2009). It has multiple functions, including regulation of collagen fibrillogenesis and maintenance of tissue integrity (Weber *et al*. 1996, Iozzo 1997, Fairlie *et al*. 1998). Decorin is also known to exhibit high levels of expression in various normal tissues, including the uterus (Su *et al*. 2004). It can regulate multiple cellular functions because of its ability to bind to a variety of molecules in both the extracellular matrix and on the cell surface (Goldoni & Iozzo 2008, Seidler & Dreier 2008). Decorin has been recently reported to sequester multiple growth factors, such as transforming growth factor β1 (TGFβ1), and directly antagonizes several members of the receptor tyrosine kinase family (Iozzo 1997, Fairlie *et al*. 1998, Seidler & Dreier 2008), including the epidermal growth factor receptor (EGFR), the insulin-like growth factor receptor I (IGFIR) (Schaefer *et al*. 2007), and the hepatocyte growth factor receptor (MET) (Goldoni *et al*. 2009, Iozzo & Sanderson 2011). Moreover, reduced decorin within tumor stromal cells has been established as a poor prognostic factor for invasive breast cancer and in murine models of spontaneous breast cancer with mammary gland carcinogenesis (Goldoni & Iozzo 2008). Decorin is a powerful endogenous tumor repressor acting in a paracrine fashion to limit tumor growth and angiogenesis (Thomas & Liliana 2012). However, it is still unclear as to how decorin affects endometrial epithelial cells or endometrial stromal cells as part of its therapeutic effect on endometriosis.

In this study, we examined the direct effects of dienogest on the *in vitro* proliferation of the human endometrial epithelial and stromal cell lines, and evaluated how decorin contributes to this effect.

## Materials and methods

### Materials

Human recombinant decorin was purchased from R&D Systems (Minneapolis, MN, USA). The rabbit monoclonal anti-human decorin antibody (product code ab151988) used for immunoblotting and immunohistochemistry (IHC) and the mouse monoclonal anti-human β-actin antibody used for immunoblotting were purchased from Abcam (Cambridge, MA, USA). The mouse monoclonal anti-human p21 antibody (product code 554228) used for immunoblotting was purchased from BD Biosciences (San Jose, CA, USA). The rabbit monoclonal anti-human c-Met antibody (product code LS-C49950) used for immunoblotting and IHC was purchased from LifeSpan Biosciences, Inc. (Seattle, WA, USA). A goat anti-human decorin antibody used as a neutralizing antibody for decorin was purchased from R&D Systems. The BD Falcon 96-well microplates used for the cell proliferation assays were purchased from BD (Franklin Lakes, NJ, USA). A rabbit anti-progesterone receptor (PR) antibody (H-190) used in the chromatin immunoprecipitation (ChIP) assay was purchased from Santa Cruz Biotechnology.

### Patients and tissue collection

A total of 50 patients who received surgical treatment between January 2002 and July 2012 were included in this study. All patients were under treatment in the Department of Obstetrics and Gynecology of Osaka Medical College. This was a retrospective cross-sectional case–controlled study carried out on human tissue samples and was approved by the Institutional Review Board of Osaka Medical College. Written informed consent was obtained from all patients participating in the study. The inclusion criteria were: age older than 20 years and no more than 50 years at the time of the surgical procedure, the presence of regular menstrual cycles (24–35 day interval) with the exception of those treated with dienogest, a fourth-generation progestin with a potent oral progestational activity without systemic androgenic effects, the absence of any evidence of past or recent pelvic inflammatory disease, and no history of any hormonal treatment for at least 12 months at baseline. Transvaginal ultrasonography was performed for all patients and showed mainly hypoechoic cystic masses in the ovaries, and the presence of ovarian endometriomas was confirmed before surgery by magnetic resonance imaging, which showed high-intensity areas on both T1- and T2-weighted images. The main indication for pre-surgical treatment was the patient's personal preference after careful explanation of the treatment options. Tissue specimens were obtained from patients treated with dienogest at a dose of 2 mg (Dienogest group; *n*=25) for three to 5 months before surgery. Simultaneous sampling of ovarian endometrioma capsules was performed during laparoscopic surgery for adnexal masses consistent with ovarian endometrioma. The scoring of endometriosis in each case was documented according to the system of the American Society for Reproductive Medicine (ASRM 1997). The diagnosis of endometriosis was confirmed histologically. The samples obtained from the endometrioma were immediately frozen in liquid nitrogen for further RT-PCR analyses, fixed in 10% formaldehyde, and then routinely processed for paraffin embedding for a histological analysis.

### Cell lines

We used a human immortalized epithelial cell line derived from an ovarian endometrioma, EMOsis CC/TERT, and primary cultured stromal cells derived from ovarian endometrioma, HMOsis scl2, and HMOsis scl3, which were established by Dr Satoru Kyo (Kanazawa University, Japan). We also used the CRL-7566 endometriosis cell line and CRL-4003 immortalized human endometrial stromal cells, which were purchased from the American Type Culture Collection (Manassas, VA, USA). EMOsis CC/TERT cells were grown in DMEM supplemented with 10% fetal bovine serum in an atmosphere of 5% CO_2_ at 37 °C (Bono *et al*. 2012). The CRL-4003 cells were cultured in growth media (DMEM/F12 10% FBS, 1% BD Insulin, Transferrin, Selenous (ITS) +Premix Universal Culture Supplement (Catalog#354352, BD)), in an atmosphere of 5% CO_2_ at 37 °C (Lockwood & Nemerson 1993, Krikun & Mor 2004). Human stromal cells derived from ovarian endometrioma, another kind gift from Dr Satoru Kyo, were used to confirm that the CRL-4003 cells possessed characteristics similar to primary cultured endometrioid stromal cells.

### RNA extraction and semi-quantitative RT-PCR

Total RNA from homogenized tissue or from cultured EMOsis CC/TERT cells or CRL-4003 cells was obtained using the RNeasy Mini kit (Qiagen), and 2 μg were reverse-transcribed with Superscript II RNase H-reverse transcriptase (Invitrogen) using random primers according to the manufacturer's instructions. The cDNA (1 μl) was amplified using 0.1 μM of each primer, 1 U of Taq DNA polymerase (Roche Diagnostics), PCR buffer with 1.5 mM MgCl_2_, and 0.25 mM dNTPs in a 20 μl reaction volume in a PTC200 Thermal cycler (Bio-Rad Laboratories, Hercules, CA, USA). The amplification conditions were as follows: initial denaturation at 94 °C for 3 min, followed by 33 cycles comprising denaturation at 94 °C for 30 s, annealing at the optimized temperature for each set of primers for 30 s, and extension at 72 °C for 30 s. The final extension was carried out for 5 min at 72 °C. The products were analyzed on 2.0% (w/v) agarose gels stained with 0.5 mg/ml ethidium bromide (Sigma–Aldrich) and visualized under an u.v. transilluminator. The product size was approximated using a 100-bp DNA ladder (Bangalore Genei, Bangalore, India). The negative control did not contain reverse transcriptase (RT) enzyme in the reaction mixture. The primers used were as follows: decorin (*DCN*), forward: 5′-AAATATTGTGCAAGGCCCGG-3′ and reverse: 5′-TTTTGCTGCCTGAGTCATCG-3′; *ER* *α* (*ESR1*)*,* forward: 5′-AGAGATGCTCCATGCCTTTG-3′ and reverse: 5′-GCAGACAGGGAGCTGGTTCA-3′; *PGR* *,* forward: 5′-AACACGTCAGTGGGCAGATG-3′ and reverse: 5′-GCAGCAATAACTTCAGACATC-3′; *PRB*, forward: 5′-TACCTCACCTGCAGCCTTCT-3′ and reverse: 5′-GCAACAGCCAGCACAAGATA-3′; *GAPDH,* forward: 5′-AGCCACATCGCTCAGACA-3′ and reverse: 5′-GCCCAATACGACCAAATCC-3′.

The experiments were repeated at least three times.

### One-step real-time PCR

Commercially available TaqMan Gene Expression Assay kits (Applied Biosystems) were used to assess the human glyceraldehyde-3-phosphate dehydrogenase (*GAPDH*; Hs02758991_g1), human β-actin (*ACTB*; Hs01060665_g1), human *RNA18S5* (Hs01060665_g1), and human *DCN* (Hs00754870_s1) gene expression levels. All primers were designed using the Primer Express software program (version 1.0; Perkin-Elmer Applied Biosystems, Carlsbad, CA, USA) from the GeneBank database according to the manufacturer's protocol. The cDNA template was amplified in a 20 μl reaction volume containing 1× TaqMan Universal PCR Master Mix (Perkin-Elmer Applied Biosystems) and 200 nM forward and reverse primers and 100 nM TaqMan probe. Thermal cycling conditions were as follow: 95 °C for 15 s followed by 60 °C for 1 min for 45 cycles in each case during One-step real-time PCR (Perkin-Elmer Applied Biosystems). Quantification cycle (Cq) values were used as an endpoint and were defined as the PCR cycle number during which the fluorescence generated by the amplification crossed the threshold. The reproducibility of the assay was tested for *ACTB*, *RNA18S5*, and *GAPDH* transcripts by calculating the coefficient of variation (CV) of repeated amplifications of the same samples, both in the same PCR and in different reactions. The average CV values were 10.9, 19.2, and 4.1%, respectively, for these transcripts. Therefore, *GAPDH* was selected as the endogenous reference gene for this study. Expression levels of *DCN* mRNA were normalized to *GAPDH* mRNA, calculated from the triplicate of CT values using the ΔΔCT method and expressed relative to one of the specimens that was assigned the value 1.

### Proliferation assay

The EMOsis CC/TERT cells in 10% fetal bovine serum and CRL-4003 cells in growth media were seeded in 96-well plates at a density of 2×10^4^ cells per well. The cells were then incubated for 48 h in the absence or presence of 0.5, 1.5, 3, or 6 μg/ml of decorin or 50, 100, 200, or 500 nM of dienogest. The concentrations of dienogest that exhibited antiproliferative effects on cells were within the range of the serum dienogest concentrations achieved with the dose (2 mg/day) used to treat endometriosis (10 and 140 nmol/l), as indicated in the previous study by Foster and Wilde (1998). CellTiter 96 AQueous (MTS) One Solution reagent (Promega) was added to each well, and the absorbance was recorded at a wavelength of 490 nm using a Corona SH-1000 lab absorbance microplate reader (Corona Electric Co., Inc., Ibaraki, Japan). The cell numbers were then calculated using a standard curve correlating the absorbance with the cell number counted under a microscope. All experiments were carried out in quadruplicate, and the cell viability was expressed as the ratio of the number of viable cells with decorin or dienogest stimulation to that of cells without stimulation.

### Western blot analysis

EMOsis CC/TERT cells and CRL-4003 cells treated with or without progesterone or dienogest were washed twice with ice-cold PBS and lysed using Pierce RIPA Buffer (Thermo Fisher Scientific, Waltham, MA, USA). Equal amounts of whole-cell protein lysates were separated by SDS–PAGE and electrotransferred onto nitrocellulose membranes. Nonspecific antigen sites were blocked with 10% BSA in 1× Tris-buffered saline for 1 h. The western blot analyses were performed with primary antibodies against decorin (1:250 dilution), c-Met (1:1000 dilution), p21 (1:500 dilution), or β-actin (1:1000 dilution) overnight at 4 °C. The immunoreactive bands in the immunoblots were visualized with a horseradish-peroxidase-coupled goat anti-rabbit immunoglobulin using an enhanced chemiluminescence western blotting system (ECL Plus, GE Healthcare Life Sciences, Pittsburgh, PA, USA). Additional membranes were analyzed by chemiluminescence before incubation with the primary antibody to confirm that the reactive band observed in the immunoblotting corresponded to a protein recognized specifically by the primary antibody.

The experiment was performed three times, and the ratio of cell cycle distribution was expressed as the mean±s.d.

### Flow cytometry

To analyze the cell cycle distribution, CRL-4003 cells and EMOsis CC/TERT cells were plated on six-well plates at a density of 2×10^5^ cells per well, then the EMOsis cc/TERT cells were cultured in 10% fetal bovine serum and the CRL-4003 cells were cultured in growth media until they reached 70–80% confluence. The cells were harvested after being incubated for 24 h in the absence or presence of 3 or 6 μg/ml of decorin or 100 or 500 μM of dienogest, then the cell proliferation was evaluated by measuring the distribution of the cells in the different phases of the cell cycle by flow cytometry using the Cycle TEST PLUS DNA Reagent kit (BD Pharmingen, San Diego, CA, USA), which is based on the measurement of the DNA content of nuclei labeled with propidium iodide, according to the manufacturer's instructions. Briefly, cells were trypsinized (250 μl of trypsin buffer) for 10 min at room temperature, and then a trypsin inhibitor (200 μl) and RNase buffer were added and allowed to react for 10 min at room temperature. Finally, propidium iodide staining solution (200 μl) was added and incubated for 10 min in the dark on ice. Samples were immediately analyzed on the EC800 flow cytometer (Sony Biotechnology Inc., Champaign, IL, USA). The FlowJo version 9 software program (Tree Star, Inc., Ashland, OR, USA) was used for the cell cycle analysis. The experiment was carried out three times, and the ratio of cell cycle distribution was expressed as the mean±s.d.

### ELISA

EMOsis cc/TERT and CRL-4003 cells were seeded at a density of 1×10^6^ cells per well in six-well plates and cultured in growth media until they reached 70–80% confluence. The cells were then starved for 16 h. The culture supernatants were collected after being incubated for 48 h with a vehicle (PBS) or 10, 100, or 500 nM of dienogest, and the level of decorin was assayed with 100 μl of cell-free culture supernatant using the Decorin Human ELISA kit (Abcam) according to the manufacturer's instructions. Absorbance was read at a wavelength of 450 nm using the Corona SH-1000 lab absorbance microplate reader (Corona Electric Co. Inc.). The sample concentrations were determined via interpolation based on the standard curve. The assay was performed three times, and the colony number was expressed as the mean±s.d.

### ChIP assays

The ChIP assay was conducted using the ChIP-IT Express ChIP kit (ACTIVE MOTIF, Carlsbad, CA, USA) according to the manufacturer's protocol. Briefly, 1×10^7^ EMOsis cc/TERT and CRL-4003 cells were incubated in growth medium. After overnight incubation, formaldehyde (37%) was directly added to the culture at a final concentration of 1%, and the cells were incubated for 15 min at 37 °C to cross-link the protein to DNA. The cells were pelleted and resuspended in 600 μl of lysis buffer supplemented with 3 μl of protease inhibitor cocktail and 50 mM phenylmethylsulfonyl fluoride, and were incubated on ice for 10 min. The nuclear fraction was resuspended in shearing buffer (supplemented with 1.05 μl protease inhibitor cocktail), and was sonicated with a sonicator. The sheared chromatin solution was used for each ChIP assay with 3 μg anti-PR antibody (H-190, Santa Cruz Biotechnology) or rabbit IgG as a negative control. After that, the 1157∼666-bp region of the decorin promoter encompassing the progesterone response element (PRE) that was predicted by the Matinspector software program (Genomatix Software GmbH, München, Germany) was amplified by conventional PCR using the following primers: F: 5′-AAATATTGTGCAAGGCCCGG-3′ and R: 5′-TTTTGCTGCCTGAGTCATCG-3′. All of the ChIP assays were repeated at least three times with similar results, and representative results for a conventional PCR are shown.

### siRNA transfection (*DCN* siRNA)

siRNA specific for decorin and the scrambled control were purchased from Invitrogen. The cells were transfected using the Lipofectamine reagent (Invitrogen) according to the manufacturer's instructions. Briefly, oligomer–Lipofectamine plus complexes were prepared as follows. A total of 20 pmol of siRNA oligomer were diluted in 50 μl of Opti-MEM (Invitrogen), and the Lipofectamine plus solution was mixed gently before use, after which a 1-μl aliquot was diluted in 50 μl of Opti-MEM, mixed gently, and incubated for 5 min at room temperature. The diluted oligomer was then combined with the diluted Lipofectamine plus, mixed gently, and incubated for another 20 min at room temperature, and the oligomer–Lipofectamine plus complexes were added to each well containing cells and medium and mixed gently by rocking the plate back and forth. The cells were subsequently incubated at 37 °C in a CO_2_ incubator for 24 h and prepared for each assay.

### IHC and scoring

The endometrioma samples were fixed in formalin and embedded in paraffin. Deparaffinized and rehydrated sections (4 μm) were then autoclaved in 0.01 mol/l citrate buffer (pH 6.0) for 15 min at 121 °C for antigen retrieval. The endogenous peroxidase activity was blocked with a 0.3% solution of hydrogen peroxide in methanol for 30 min, and the sections were subsequently incubated at 4 °C for 12 h with anti-decorin rabbit antibodies (1:100 dilution; Abcam) or anti-Met rabbit antibodies (1:100 dilution; LifeSpan BioSciences). The sections were then washed with 1× PBS (PBS) and incubated with Histofine simple stain MAX PO (multi; Nichirei) for 30 min at room temperature. Finally, the sections were washed with 1× PBS, and the signals were visualized after incubation with H_2_O_2_/diaminobenzidine substrate solution for 5 min. Tissue IHC staining was evaluated under a light microscope. The tissue IHC staining was analyzed blindly and independently by two examiners using a four-point semi-quantitative scale for intensity: 3+ (very strong), 2+ (strong), 1+ (moderate/weak), and 0 (no staining) (Cawthorn *et al*. 2012). In cases where there was a disagreement between the examiners, the scores were reviewed together and a consensus was reached.

### Statistical analyses

All experiments were carried out in triplicate, except for the cell proliferation assay. The statistical calculations were performed using the StatView statistical software package (SAS Institute, Cary, NC, USA), and the statistical significance of each difference was determined using the Kruskal–Wallis and Mann–Whitney *U*-test, or a paired *t*-test, as appropriate. A value of *P*<0.05 was considered to be statistically significant.

## Results

### Progesterone treatment promoted the expression and secretion of decorin in the EMOsis cc/TERT and CRL-4003 cells

**Figure 1:**
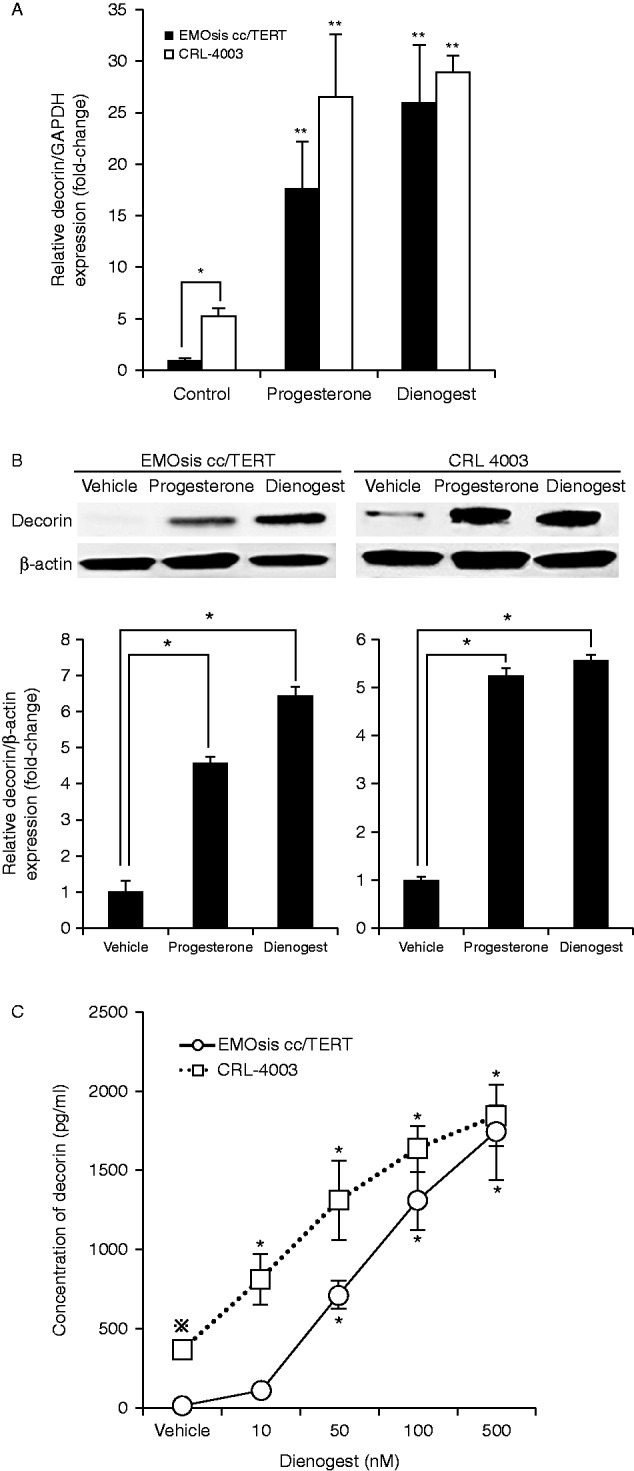
(A) Progesterone treatment promoted *DCN* mRNA expression in EMOsis cc/TERT cells and CRL-4003 cells. The mRNA expression of decorin was determined by real-time PCR from the total RNA obtained from CRL-4003 cells and EMOsis cc/TERT cells cultured with or without decorin or dienogest for 12 h. The mRNA levels of decorin were normalized to those of *GAPDH* mRNA. The data were processed by the comparative Ct method and expressed as a fold-increase relative to the basal transcription level in the control. Bars indicate the s.e.m. Significant differences are indicated by asterisks. *P<0.05, **P<0.01. (B) Progesterone treatment promoted the expression of decorin in EMOsis cc/TERT cells and CRL-4003 cells. CRL-4003 cells and EMOsis cc/TERT cells were harvested and used to prepare cell lysates after treatment with or without dienogest or progesterone for 24 h. The lysates were subjected to SDS–PAGE and blotted with anti-decorin (upper panel) or anti-β-actin (lower panel) antibodies. The lower panel shows the densitometric quantification of the western blot analysis normalized to β-actin expression and expressed as a fold-increase relative to the basal transcription level in the control. The mean ±s.d. of three determinations is shown. *P<0.05. (C) Progesterone treatment promoted the secretion of decorin in EMOsis cc/TERT cells and CRL-4003 cells. CRL-4003 cells and EMOsis cc/TERT cells were treated with dienogest at various concentrations for 48 h, and the concentrations of decorin produced by the EMOsis cc/TERT and CRL-4003 cells were measured using the Decorin (DCN) Human ELISA kit. The data are expressed as the mean±s.d. (*N*=5). * indicates a significant (*P*<0.05) difference compared with the untreated control group, while ⋇ indicates a significant (*P*<0.05) difference compared with untreated control EMOsis cc/TERT cells.

EMOsis cc/TERT cells, which are immortalized human epithelial cells derived from an ovarian endometrioma, and CRL-4003 cells, which are immortalized human endometrial stromal cells, were treated with a vehicle (PBS), 1.5 μg/ml progesterone, or 100 nmol/l dienogest for 12 h. The CV values of the mRNA expression (Cq) in all cell samples tested for *GAPDH*, *ACTB*, and *RNA18S5* were 4.1, 10.9, and 19.2% respectively. Therefore, normalization against GAPDH alone met the standards described in the MIQE guidelines (Bustin *et al*. 2009). The *DCN* mRNA expression was significantly increased in both the EMOsis cc/TERT cells and CRL-4003 cells treated with progesterone or dienogest compared with the expression in the control cells by performing, determined by real-time PCR (*P*<0.05) (Fig. 1A).

Then, we ascertained whether progesterone and dienogest promoted decorin synthesis in the EMOsis cc/TERT and CRL-4003 cells by performing a western blot analysis. The expression levels of the decorin protein in EMOsis cc/TERT cells and CRL-4003 cells treated with progesterone or dienogest were significantly increased compared with those for the untreated cells (*P*<0.05) (Fig. 1B).

We measured the concentrations of decorin produced by the EMOsis cc/TERT and CRL-4003 cells using the Decorin Human ELISA kit. The decorin concentration significantly increased in a dose-dependent manner following dienogest treatment (*P*<0.05) (Fig. 1C), indicating that progestin stimulation promoted decorin secretion in both cell lines. Interestingly, the concentration of decorin in the cultured medium of untreated CRL-4003 cells was higher than that observed in the cultured medium of EMOsis cc/TERT cells (*P*<0.05) (Fig. 1C).

### Dienogest inhibits the proliferation of EMOsis cc/TERT and CRL-4003 cells via the effects on decorin

**Figure 2:**
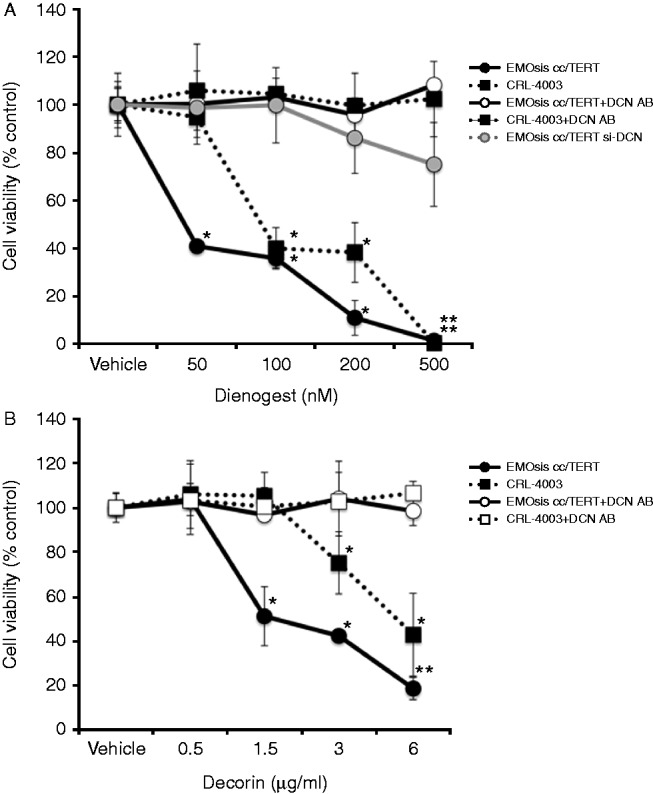
(A) Progesterone treatment inhibits the proliferation of EMOsis cc/TERT cells and CRL-4003 cells in a decorin-dependent manner. CRL-4003 cells, EMOsis cc/TERT cells, and EMOsis cc/TERT cells transfected with the siRNA specific for decorin (si-DCN) were treated with dienogest at various concentrations with or without the decorin-neutralizing antibody (5 μg/ml) for 48 h, and the proliferation was measured by the MTS assay. (B) Decorin treatment inhibits the proliferation of EMOsis cc/TERT cells and CRL-4003 cells in a decorin-dependent manner. CRL-4003 cells and EMOsis cc/TERT cells were treated with decorin at various concentrations with or without a decorin-neutralizing antibody (5 μg/ml) for 48 h, and the proliferation was measured by the MTS assay. The data are expressed as the means±s.d. (*n*=5), and * indicates a significant (*P*<0.05) difference compared with the untreated control.

We examined whether dienogest and decorin inhibited the proliferation of EMOsis cc/TERT cells and CRL-4003 cells. The growth of the EMOsis cc/TERT cells and CRL-4003 cells was inhibited in a dose-dependent manner by dienogest (Fig. 2A). However, these effects were canceled by transfection with the *DCN* siRNA or the addition of a decorin-neutralizing antibody (Fig. 2A). Moreover, the growth of the EMOsis cc/TERT cells and CRL-4003 cells was also inhibited by decorin in a dose-dependent manner (Fig. 2A), and these findings were also canceled by the addition of a decorin-neutralizing antibody (Fig. 2B). These results indicate that dienogest and decorin possess cytostatic effects, and that dienogest apparently inhibits cell growth via its effects on decorin.

### Progesterone and dienogest play crucial roles in stimulating the *DCN* gene expression in EMOsis cc/TERT and CRL-4003 cells

**Figure 3:**
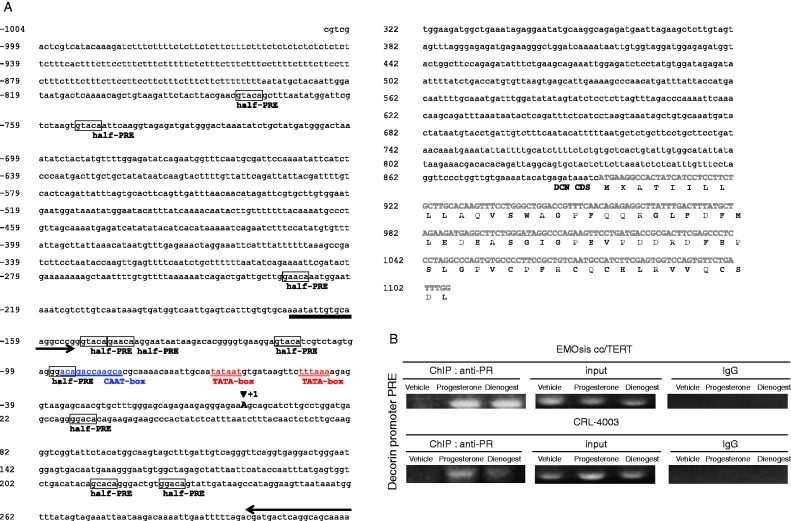
Dienogest and progesterone play a crucial role in stimulating the *DCN* gene expression in EMOsis cc/TERT and CRL-4003 cells. (A) The half-PRE island is located in the Decorin promoter. The nucleotide sequence and putative regulatory elements of the 5′-flanking region of the human *DCN* gene are shown. The coding sequence of the human *DCN* gene is indicated by bold and capital letters. The blue underlined letters and red double underlined letters indicate the CAAT-box and TATA-box respectively. The large A residue at position +1 with an arrowhead above indicates the transcriptional initiation site. Boxed letters indicate the half-PRE sites. The arrows indicate the primer used for PCR amplification in the ChIP assay. (B) Progesterone and dienogest directly stimulated decorin gene expression. The ChIP assay, performed as described in the Materials and methods section, showed the expression of the decorin promoter in the control medium or progesterone or dienogest-stimulated EMOsis cc/TERT and CRL-4003 cells. An anti-PR antibody (Santa Cruz Biotechnology) was used for immunoprecipitation. The input DNA represents PCR products from chromatin pellets before immunoprecipitation. IgG was used as the negative control, and normal rabbit anti-IgG antibodies were used. The PCR primer was designed as described in the Materials and methods section.

To directly evaluate whether progesterone and dienogest play a role in stimulating decorin expression, we evaluated whether the PR was recruited to the promoter of the *DCN* gene by performing the ChIP assay, as shown in Fig. 3A. The decorin promoter region between −1010 bp and −679 bp contains several half-PREs (hPRE), two TATA-boxes, and one CAAT-box (Santra *et al*. 1994). Therefore, ChIP primers for the decorin promoter region were designed to cover the region from −1157 bp and −666 bp. The EMOsis cc/TERT and CRL-4003 cells were exposed to a vehicle (PBS), 1.5 μg/ml progesterone, or 100 nmol/l dienogest overnight, and then processed for the ChIP assay. Progesterone and dienogest induced the binding of the PR to the hPRE-binding site of the decorin promoter in both EMOsis cc/TERT and CRL-4003 cells (Fig. 3B). These results indicate that progesterone and dienogest play a crucial role in stimulating *DCN* gene expression in EMOsis cc/TERT cells and CRL-4003 cells.

### Progesterone and dienogest induce cell cycle arrest via the actions of decorin by promoting p21 production and suppressing the expression of MET in EMOsis cc/TERT and CRL-4003 cells

**Figure 4:**
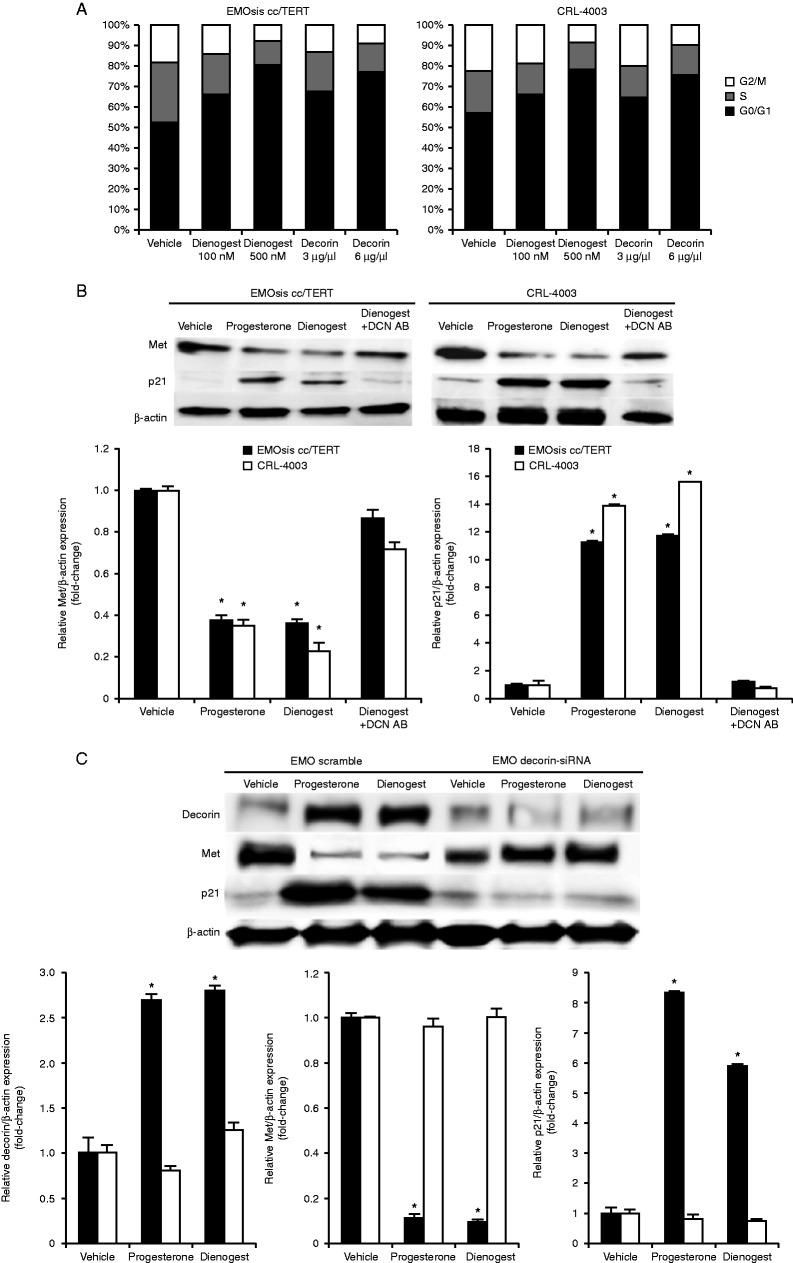
Dienogest induces cell cycle arrest in EMOsis cc/TERT cells and CRL-4003 cells by promoting p21 production and affects MET expression. (A) Inhibition of cell cycle progression by decorin and dienogest. EMOsis cc/TERT cells and CRL-4003 cells were exposed to 100 or 500 nmol/l dienogest or 3 or 6 μg/ml decorin overnight, or were left untreated, and then processed for PI flow cytometry. The cell cycle characteristics (G0–G1: S: G2–M%) of EMOsis cc/TERT cells were as follows: control (52.64±0.67: 29.45±0.92: 17.91±1.08), 100 nM dienogest (66.39±0.18*: 19.33±0.36*: 14.28±1.81), 500 nM dienogest (80.38±0.57*: 12.14±0.32*: 7.48±0.37*), 3 mg/ml decorin (67.59±0.95*: 19.8±0.04*: 12.61±0.83), and 6 mg/ml decorin (77.21±0.26*: 14.51±0.64*: 8.28±0.56*) and CRL-4003 cells: control (57.23±0.10: 20.46±0.63: 22.31±0.71), 100 nM dienogest (66.57±0.38*: 15.23±0.09: 18.2±0.26), 500 nM dienogest (78.07±0.69*: 13.61±0.17*: 8.32±0.91*), 3 mg/ml decorin (65.05±0.50*: 15.22±0.75: 19.73±0.27), and 6 mg/ml decorin (75.31±0.47*: 15.28±1.08*: 9.41±0.29*). The data are expressed as the means±s.d. (*N*=5), and * indicates a significant (*P*<0.05) difference compared with the untreated control. The panel shows the proportion of cells in the G0/G1 phase (black), S phase (gray), and G2/M phase (white). (B) The expression of p21 was increased and the expression of MET was decreased by dienogest and progesterone in a decorin-dependent manner. Proteins were extracted from CRL-4003 cells and EMOsis cc/TERT cells after treatment with 100 nmol/l dienogest, 100 nmol/l progesterone, or 100 nmol/l dienogest with or without 5 μg/ml of a decorin-neutralizing antibody for 24 h. The western blot analysis was performed with anti-Met antibodies, anti-p21 antibodies, and anti-β-actin antibodies. The lower panel shows the densitometric quantification of the western blot analysis normalized to the β-actin expression and expressed as a fold-increase relative to the basal transcription level in the control. The mean±s.d. of three determinations is shown. *P<0.05 (C) Decorin and p21 expression is increased and MET expression is decreased by dienogest and progesterone in a decorin-dependent manner. Proteins were extracted from CRL-4003 cells, EMOsis cc/TERT cells, and EMOsis cc/TERT cells transfected with *DCN* siRNA cultured with or without progesterone or dienogest for 24 h. The western blot analysis was performed with anti-decorin antibodies, anti-p21 antibodies, anti-Met antibodies, and anti-β-actin antibodies. The lower panel shows the densitometric quantification of the western blot analysis normalized to the β-actin expression and expressed as a fold-increase relative to the basal transcription level in the control. The mean±s.d. of three determinations is shown. *P<0.05

To determine whether the anti-proliferative effects of decorin synthesis promoted by dienogest were accompanied by an effect on the cell cycle profile, EMOsis cc/TERT cells and CRL-4003 cells were treated with a vehicle (PBS), dienogest, or decorin for 16 h, and then processed for PI flow cytometry to determine the distribution of cells in the various stages of the cell cycle. As shown in Fig. 4A, cells treated with either dienogest or decorin exhibited a significant increase in G0/G1 phase cells and a reduction in S and G2–M phase cells compared with untreated cells of both types and the effects of dienogest and decorin were dose-dependent (*P*<0.05). Therefore, the dienogest and decorin treatment induced cell cycle arrest in EMOsis cc/TERT cells and CRL-4003 cells.

Then, we examined the expression of *CDKN1A* in EMOsis cc/TERT and CRL-4003 cells treated with progesterone or dienogest. As shown in Fig. 4B, treatment with progesterone and/or dienogest led to a significant decrease in the expression of MET and a significant increase in the expression of *CDKN1A* in both the EMOsis cc/TERT and CRL-4003 cells compared with that observed in untreated cells (*P*<0.05 for both). However, these effects were canceled by the addition of a decorin-neutralizing antibody as well as transfection with the *DCN* siRNA (Fig. 4C). These observations indicate that decorin directly increased the expression of *CDKN1A* in both the EMOsis cc/TERT and CRL-4003 cells.

### Dienogest treatment promoted the expression of the decorin in ovarian endometrioma

Based on the findings that progesterone and dienogest have anti-proliferative effects via the upregulation on decorin, we sought to examine the clinical relevance of decorin in patients with endometrioma. A total of 50 females who underwent surgical treatment for endometrioma were evaluated in this study. They were divided into two groups, 25 females who did not receive any medical treatment before surgery (control group) and 25 females who received dienogest (a fourth-generation progestin) (Table 1). There were no relevant group differences in terms of the age, VAS score, cyst size, or ASRM score at baseline. The use of concomitant medications recorded in patient-maintained diaries, including analgesic medications for endometriosis, did not differ significantly between the groups at baseline. The mean (±s.d.) VAS score at baseline was 35.6±20.4 mm in the control group and 48.3±26.1 mm in the dienogest group. The mean (±s.d.) ASRM score at baseline was 39.5±17.9 in the control group and 38.7±20.3 in the dienogest group. The mean (±s.d.) cyst size at baseline was 60.7±14.9 mm in the control group and 57.5±15.5 mm in the dienogest group. There were no significant differences between the two groups. However, the mean (±s.d.) post-treatment cyst size was 44.7±12.8 mm in the dienogest group, which was significantly different from the pre-treatment cyst size in this group (*P*=0.0002).

**Figure 5:**
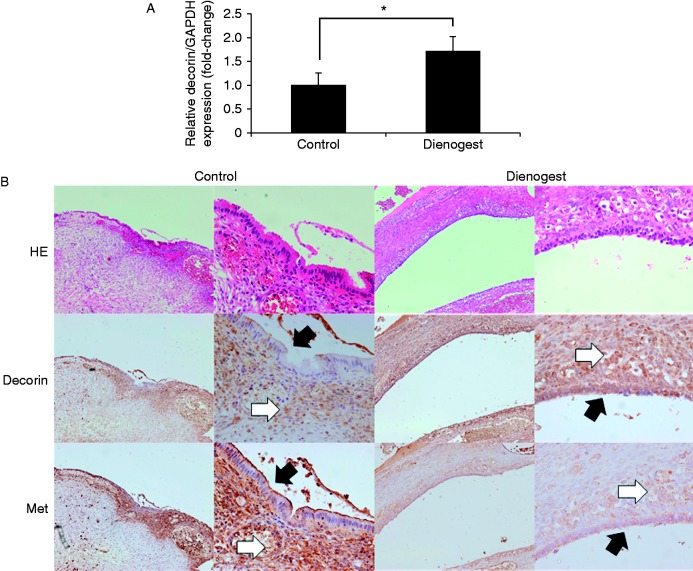
Progesterone treatment promoted the expression of decorin in ovarian endometrioma. (A) *DCN* mRNA expression in patients with ovarian endometrioma. A total of 50 cases were selected for the study, and RNA was extracted from surgically obtained endometrioma tissue samples from the control group (*n*=25) and dienogest group (*n*=25). The mRNA levels of decorin were measured by real-time PCR and normalized to those of *GAPDH* mRNA. The data were processed by the comparative Ct method and expressed as a fold-increase relative to the basal transcription level in the control. Bars indicate the s.e.m. Significant differences are indicated by an asterisk. *P<0.05 (B) Decorin and MET expression in ovarian endometrioma. Representative examples of immunohistochemical sequential sections stained with decorin and MET among the ovarian endometrioma samples obtained from endometriosis patients who underwent oophorectomy are shown. A representative section from the no-preoperative-treatment control group is on the left, and a representative section from the dienogest preoperative treatment group is on the right. The black filled arrows indicate the epithelial cells and the white filled arrows indicate the stromal cells.

We investigated whether pre-surgical treatment with dienogest attenuated the expression of decorin in the ectopic endometrium. The CV values of *GAPDH*, *ACTB*, and *RNA18S5* mRNA expression (Cq) in all patient samples tested were 4.1, 10.9, and 19.2% respectively. Therefore, normalization against GAPDH alone met the standards described in the MIQE guidelines (Bustin *et al*. 2009). The endometrioma samples obtained from patients who received dienogest treatment showed significantly higher expression of *DCN* mRNA compared with the control tissues using real-time PCR (Fig. 5A). Moreover, we confirmed that the expression of decorin in the ectopic endometrium of patients in the dienogest-treated group was significantly higher than that observed in the control tissue by IHC analysis (staining intensity of epithelial cells: control, 0.17±0.38; dienogest, 2.39±0.61; staining intensity of stromal cells: control, 1.17±0.38; dienogest, 2.94±0.24; *P*<0.05). We also confirmed that the expression of MET was significantly lower in the ectopic endometrium of patients in the dienogest-treated group (staining intensity of epithelial cells: control, 2.33±0.69; dienogest, 0.94±0.64; staining intensity of stromal cells: control, 2.67±0.59; dienogest, 0.78±0.65; *P*<0.05) (Fig. 5B).

## Discussion

In this study, we showed that the endometriotic cells in patients treated with dienogest had a higher *DCN* mRNA expression levels than those in untreated patients. A previous report has described the inhibition of the proliferation of immortalized human endometrial epithelial cells by dienogest via suppression of *CCND1* gene expression (Shimizu *et al*. 2009); however, this did not occur in endometrial stromal cells. Elsewhere, it has been reported that dienogest showed antiproliferative effects on both human eutopic and ectopic endometrial stromal cells (Okada *et al*. 2001, Fu *et al*. 2008). However, previous studies have not revealed how progesterone directly affects the proliferation of the human endometriosis cells. In this study, we demonstrated, for the first time, to our knowledge, that progesterone and dienogest have anti-proliferative effects via their effects on decorin. Moreover, we demonstrated that this was the case not only for human endometriotic epithelial cells, but also for endometrial stromal cells. In the current study, we used CRL-4003 cells, an immortalized human endometrial stromal cell line, and not ectopic endometrioid stromal cells. However, we confirmed that the CRL-4003 cells were useful in identifying both the hormonal status (estrogen receptor (ER) and progesterone receptor (PR)) and expression of *DCN* mRNA treated with dienogest in primary cultured endometriotic stromal cells (HMOsis scl2 and HMOsis scl3) and the CRL-7566 endometriosis cell line (Supplementary Figure 1, see section on supplementary data given at the end of this article.). Our findings indicate that both CRL-4003 cells and ectopic endometrioid stromal cells have identical characteristics for testing the effects of dienogest and decorin on stromal cells, with respect to the convenience of experimentation.

We demonstrated that progesterone and dienogest directly induced the binding of the PR to the PRE-binding site of the decorin promoter in endometriotic epithelial cells and endometrial stromal cells by the ChIP assay, and that decorin was synthesized by the endometriotic epithelial cells and endometrial stromal cells. Decorin induced cell cycle arrest in endometrial epithelial cells and endometrial stromal cells. It has previously been shown that decorin induces cell cycle arrest by inducing the cyclin-dependent protein kinase inhibitor, p21^Waf1/Cip1^, decreasing the activity and abundance of multiple cyclins, and activating pro-apoptotic pathways (Iozzo & Sanderson 2011). We clarified that the signaling pathway involved decorin and p21 synthesis, as the dienogest-induced increase in the expression of p21 in the endometriotic epithelial cells and stromal cells was significantly reduced by both the addition of a decorin-neutralizing antibody and transfection with the *DCN* siRNA. Consequently, our results indicated that the direct antiproliferative effects of progesterone and dienogest occur via the induction of G_0_/G_1_ cell cycle arrest via decorin, and that increased levels of progesterone and dienogest lead to the upregulation of p21 synthesis via the upregulation of decorin in human endometriotic epithelial cells and endometrial stromal cells.

The prototypic members of the small leucine-rich proteoglycan family include not only decorin, but also biglycan, lumican, and fibromodulin (Kresse *et al*. 1993, Iozzo 1998, Neame & Kay 2000). The patterns of expression of these prototype members of the small leucine-rich proteoglycan family in the uterus were found to be dependent on estrogen or progesterone in experiments carried out using mouse models (Salgado *et al*. 2011). Although we could not evaluate whether the other small leucine-rich proteoglycans, including biglycan, lumican, and fibromodulin, affect the endometriotic epithelial cells or endometrial stromal cells (contributing to the therapeutic effect on endometriosis), decorin is known to have the most potent tumor-suppressive effects compared with other molecules.

There have been recent reports of the anti-tumor effects of decorin on various malignant tumors, including uterine cervical carcinoma cells (Grant *et al*. 2002), ovarian cancer cells (Nash *et al*. 1999), colon carcinoma cells (Santra *et al*. 1995), breast cancer cells (Reed *et al*. 2005), and pancreatic cancer cells (Koninger *et al*. 2004). Previous results have indicated that decorin is a powerful inhibitor of tumor angiogenesis in several malignant cell lines (Grant *et al*. 2002). Endometriosis has been shown to be highly dependent on angiogenesis for maintenance and growth (Nap *et al*. 2004). Increased levels of decorin lead to the downregulation of MET, which is known to be a hepatocyte growth factor receptor, an established mediator of malignant transformation, invasion, and metastasis (Zhang *et al*. 2003). In this study, decorin suppressed the expression of MET in endometriotic epithelial cells and endometrial stromal cells, which contributed to its therapeutic effects on endometriosis.

These findings indicated that the estrogen-independent anti-proliferative effects of decorin on endometriotic epithelial cells and endometrial stromal cells may contribute to the effectiveness against endometriosis, without causing hypoestrogenic side effects, such as bone loss and hot flushes. Moreover, decorin has minimal toxicity, because it is a naturally ubiquitous proteoglycan (Nap *et al*. 2004). Further examinations will be necessary to determine the optimum strategy for obtaining therapeutic benefits of decorin treatment in patients with endometriosis.

In summary, we demonstrated that progesterone and dienogest inhibit the proliferation of immortalized human endometriosis epithelial cells and stromal cells via the upregulation of decorin. Moreover, we demonstrated that decorin has significant antiproliferative effects on human endometriotic epithelial cells and endometrial stromal cells due to its actions in inducing cell cycle arrest via p21 synthesis in endometriotic tissues.

## Supplementary data

This is linked to the online version of the paper at http://dx.doi.org/10.1530/JOE-14-0393.

## Author contribution statement

Y Ono conducted clinical evaluation, contributed to sample collection and manuscript writing, and performed most of the experiments. Y Terai, A Tanabe, and M Hayashi designed this study, analyzed data, and wrote the manuscript. Y Terai, A Hayashi, and S Kyo were involved in clinical material collection and analysis. Y Yamashita was involved in study design and data interpretation. Y Terai and M Ohmichi were involved in study conception and design, data analysis, drafting the article, and its final edition. All authors gave their final approval of the submitted version.

## Supplementary Material

Supplementary Figure

## Figures and Tables

**Table 1:** Patient characteristics according to the treatment group

**Characteristics**	**Control**	**Dienogest**	***P*** **value**
Number of patients	25	25	NS
Age (years, mean) (±s.d.)	35.5±6.23	33.1±6.86	NS
Duration of drug administration (weeks, mean)	–	21.6±11.5	–
ASRM score (mean)	39.5±17.9	38.7±20.3	NS
Pre-treatment cyst size (maximum diameter mm, mean)	60.7±14.9	57.5±15.5	NS
Post-treatment cyst size (maximum diameter mm, mean)	–	44.7±12.8^a^	–
Pre-treatment pelvic pain VAS (mm, mean)	35.6±20.3	48.3±20.3	NS
Post-treatment pelvic pain VAS (mm, mean)	–	20.6±15.1	–

ASRM score, American Society for Reproductive Medicine score; VAS score, visual analog scale score; *P* value between the pre- and post-treatment cyst size: 0.0002.

a Paired *t-*test.

## References

[bib1] American Society for Reproductive Medicine (1997). Revised American Society for Reproductive Medicine classification of endometriosis. Fertility and Sterility.

[bib2] Bono Y, Kyo S, Takakura M, Maida Y, Mizumoto1 Y, Nakamura M, Nomura K, Kiyono T, Inoue M (2012). Creation of immortalised epithelial cells from ovarian endometrioma. British Journal of Cancer.

[bib3] Brown J, Pan A, Hart RJ (2010). Gonadotrophin-releasing hormone analogues for pain associated with endometriosis. Cochrane Database of Systematic Reviews.

[bib4] Bustin SA, Benes V, Garson JA, Hellemans J, Huggett J, Kubista M, Mueller R, Nolan T, Pfaffl MW, Shipley GL (2009). The MIQE guidelines: minimum information for publication of quantitative real-time PCR experiments. Clinical Chemistry.

[bib5] Cawthorn TR, Moreno JC, Dharsee M, Tran-Thanh D, Ackloo S, Zhu PH, Sardana G, Chen J, Kupchak P, Jacks LM (2012). Proteomic analyses reveal high expression of decorin and endoplasmin (HSP90B1) are associated with breast cancer metastasis and decreased survival. PLoS ONE.

[bib6] Cosson M, Querleu D, Donnez J, Madelenat P, Konincks P, Audebert A, Manhes H (2002). Dienogest is as effective as triptorelin in the treatment of endometriosis after laparoscopic surgery: results of a prospective, multicenter, randomized study. Fertility and Sterility.

[bib7] Danielson KG, Baribault H, Holmes DF, Graham H, Kadler KE, Iozzo RV (1997). Targeted disruption of decorin leads to abnormal collagen fibril morphology and skin fragility. Journal of Cell Biology.

[bib8] Eskenazi B, Warner ML (1997). Epidemiology of endometriosis. Obstetrics and Gynecology Clinics of North America.

[bib9] Fairlie DP, West ML, Wong AK (1998). Towards protein surface mimetics. Current Medicinal Chemistry.

[bib10] Felice P, Daniela H, Christian S, Thomas F, Christoph G, Stefano L, Lucia L, Thomas S (2012). Reduced pelvic pain in women with endometriosis: efficacy of long-term dienogest treatment. Archives of Gynecology and Obstetrics.

[bib11] Foster RH, Wilde MI (1998). Dienogest. Drugs.

[bib12] Fu L, Osuga Y, Morimoto C, Hirata T, Hirota Y, Yano T, Taketani Y (2008). Dienogest inhibits BrdU uptake with G0/G1 arrest in cultured endometriotic stromal cells. Fertility and Sterility.

[bib13] Giudice LC, Kao LC (2004). Endometriosis. Lancet.

[bib14] Goldoni S, Iozzo RV (2008). Tumor microenvironment: modulation by decorin and related molecules harboring leucine-rich tandem motifs. International Journal of Cancer.

[bib15] Goldoni S, Humphries A, Nystrom A, Sattar S, Owens RT, Mc-Quillan DJ, Ireton K, Iozzo RV (2009). Decorin is a novel antagonistic ligand of the Met receptor. Journal of Cell Biology.

[bib16] Grant DS, Yenisey C, Rose RW, Tootell M, Santra M, Iozzo RV (2002). Decorin suppresses tumor cell-mediated angiogenesis. Oncogene.

[bib17] Guo SW (2009). Recurrence of endometriosis and its control. Human Reproduction Update.

[bib18] Horie S, Harada T, Mitsunari M, Taniguchi F, Iwabe T, Terakawa N (2005). Progesterone and progestational compounds attenuate tumor necrosis factor α-induced interleukin-8 production via nuclear factor κ B inactivation in endometriotic stromal cells. Fertility and Sterility.

[bib19] Iozzo RV (1997). The family of the small leucine-rich proteoglycans: key regulators of matrix assembly and cellular growth. Critical Reviews in Biochemistry and Molecular Biology.

[bib20] Iozzo RV (1998). Matrix proteoglycans: from molecular design to cellular function. Annual Review of Biochemistry.

[bib21] Iozzo RV, Sanderson RD (2011). Proteoglycans in cancer biology, tumor microenvironment and angiogenesis. Journal of Cellular and Molecular Medicine.

[bib22] Irahara M, Harada T, Momoeda M, Taketani Y (2007). Hormonal and histological study on irregular genital bleeding in patients with endometriosis during treatment with dienogest, a novel progestational therapeutic agent. Reproductive Medicine and Biology.

[bib23] Jee BC, Lee JY, Suh CS, Kim SH, Choi YM, Moon SY (2009). Impact of GnRH agonist treatment on recurrence of ovarian endometriomas after conservative laparoscopic surgery. Fertility and Sterility.

[bib24] Keene DR, San Antonio JD, Mayne R, McQuillan DJ, Sarris G, Santoro SA, Iozzo RV (2000). Decorin binds near the C terminus of type I collagen. Journal of Biological Chemistry.

[bib25] Köhler G, Faustmann TA, Gerlinger C, Seitz C, Mueck AO (2010). A dose-ranging study to determine the efficacy and safety of 1, 2, and 4 mg of dienogest daily for endometriosis. International Journal of Gynaecology and Obstetrics.

[bib26] Koninger J, Giese NA, Francesco di Mola F, Berberat P, Giese T, Esposito I, Bachem MG, Friess H (2004). Overexpressed decorin in pancreatic cancer: potential tumor growth inhibition and attenuation of chemotherapeutic action. Clinical Cancer Research.

[bib27] Kresse H, Hausser H, Schönherr E (1993). Small proteoglycans. Experientia.

[bib28] Krikun G, Mor G (2004). A novel immortalized human endometrial stromal cell line with normal progestational response. Endocrinology.

[bib29] Lockwood CJ, Nemerson Y (1993). Progestational regulation of human endometrial stromal cell tissue factor expression during decidualization. Journal of Clinical Endocrinology and Metabolism.

[bib30] Momoeda M, Harada T, Terakawa N, Aso T, Fukunaga M, Hagino H, Taketani Y (2009). Long-term use of dienogest for the treatment of endometriosis. Journal of Obstetrics and Gynaecology Research.

[bib31] Nap AW, Griffioen AW, Dunselman GA, Bouma-ter Steege JC, Thijssen VL, Evers JL, Groothuis PG (2004). Antiangiogenesis therapy for endometriosis. Journal of Clinical Endocrinology and Metabolism.

[bib32] Nash MA, Loercher AE, Freedman RS (1999). *In vitro* growth inhibition of ovarian cancer cells by decorin: synergism of action between decorin and carboplatin. Cancer Research.

[bib33] Neame PJ, Kay CJ, Iozzo RV (2000). Small leucine-rich proteoglycans. Proteoglycans, Structure, Biology and Molecular Interactions.

[bib34] Okada H, Nakajima T, Yoshimura T, Yasuda K, Kanzaki H (2001). The inhibitory effect of dienogest, a synthetic steroid, on the growth of human endometrial stromal cells *in vitro*. Molecular Human Reproduction.

[bib35] Reed CC, Waterhouse A, Kirby S, Kay P, Owens RT, McQuillan DJ, Izzo RV (2005). Decorin prevents metastatic spreading of breast cancer. Oncogene.

[bib36] Salgado RM, Favaro RR, Zorn TM (2011). Modulation of small leucine-rich proteoglycans (SLRPs) expression in the mouse uterus by estradiol and progesterone. Reproductive Biology and Endocrinology.

[bib37] Santra M, Danielson KG, Iozzo RV (1994). Structural and functional characterization of the human decorin gene promoter. Journal of Biological Chemistry.

[bib38] Santra M, Skorski T, Calabretta B, Lattime EC, Iozzo RV (1995). *De novo* decorin gene expression suppresses the malignant phenotype in human colon cancer cells. PNAS.

[bib39] Sasagawa S, Shimizu Y, Kami H, Takeuchi T, Mita S, Imada K, Kato S, Mizuguchi K (2008). Dienogest is a selective progesterone receptor agonist in transactivation analysis with potent oral endometrial activity due to its efficient pharmacokinetic profile. Steroids.

[bib40] Schaefer L, Tsalastra W, Babelova A, Baliova M, Minnerup J, Sorokin L, Grone HJ, Reinhardt DP, Pfeilschifter J, Iozzo RV (2007). Decorin-mediated regulation of fibrillin-1 in the kidney involves the insulin-like growth factor-I receptor and mammalian target of rapamycin. American Journal of Pathology.

[bib41] Seidler DG, Dreier R (2008). Decorin and its galactosaminoglycan chain: extracellular regulator of cellular function?. IUBMB Life.

[bib42] Shimizu Y, Takeuchi T, Mita S, Mizuguchi K, Kiyono T, Inoue M, Kyo S (2009). Dienogest, a synthetic progestin, inhibits the proliferation of immortalized human endometrial epithelial cells with suppression of cyclin D1 gene expression. Molecular Human Reproduction.

[bib43] Sitruk WR (2004a). New progestogens: a review of their effects in perimenopausal and postmenopausal women. Drugs & Aging.

[bib44] Sitruk WR (2004b). Pharmacological profile of progestins. Maturitas.

[bib45] Strowitzki T, Faustmann A, Christoph G, Seitz C (2010a). Dienogest in the treatment of endometriosis-associated pelvic pain: a 12-week, randomized, double-blind, placebo-controlled study. European Journal of Obstetrics, Gynecology, and Reproductive Biology.

[bib46] Strowitzki T, Marr J, Gerlinger C, Faustmann T, Seitz C (2010b). Dienogest is as effective as leuprolide acetate in treating the painful symptoms of endometriosis: a 24-week, randomized, multicentre, open-label trial. Human Reproduction.

[bib47] Su AI, Wiltshire T, Batalov S, Lapp H, Ching KA, Block D, Zhang J, Soden R, Hayakawa M, Kreiman G (2004). A gene atlas of the mouse and human protein-encoding transcriptomes. PNAS.

[bib48] Thomas N, Liliana S (2012). A guardian from the matrix. American Journal of Pathology.

[bib49] Vercellini P, Somigliana E, Viganò P, Abbiati A, Barbara G, Crosignani PG (2009). Endometriosis: current therapies and new pharmacological developments. Drugs.

[bib50] Weber IT, Harrison RW, Iozzo RV (1996). Model structure of decorin and implications for collagen fibrillogenesis. Journal of Biological Chemistry.

[bib51] Zhang YW, Su Y, Volpert OV, Vande Woude GF (2003). Hepatocyte growth factor/scatter factor mediates angiogenesis through positive VEGF and negative thrombospondin 1 regulation. PNAS.

[bib52] Zhang G, Ezura Y, Chervoneva I, Robinson PS, Beason DP, Carine ET, Soslowsky LJ, Iozzo RV, Birk DE (2006). Decorin regulates assembly of collagen fibrils and acquisition of biomechanical properties during tendon development. Journal of Cellular Biochemistry.

[bib53] Zhang G, Chen S, Goldoni S, Calder BW, Simpson HC, Owens RT, McQuillan DJ, Young MF, Iozzo RV, Birk DE (2009). Genetic evidence for the coordinated regulation of collagen fibrillogenesis in the cornea by decorin and biglycan. Journal of Biological Chemistry.

