# Supplementary material for: Decorin induced by progesterone plays a crucial role in suppressing endometriosis
Source: J Endocrinol. 2014 Nov;223(2):203–16. doi: 10.1530/JOE-14-0393 (PMC4198121; doi:10.1530/JOE-14-0393)
Supplement: Supplementary Figure [file supp_223_2_203__index.html]

Decorin induced by progesterone plays a crucial role in suppressing endometriosis — Progestin suppresses endometriosis via decorin — Supplementary Figure 

# Decorin induced by progesterone plays a crucial role in suppressing endometriosis

## Supplementary Figure

**Files in this Data Supplement:**

- Supplementary Figure 1 - (A) We evaluated the expression of ER mRNA and PR mRNA, in the primary cultured cells (HMOsis scl2 and HMOsis scl3) which were stromal cells derived from ovarian endometrioma and CRL-7566 endometriosis cell line using RT-PCR. HMOsis scl2, HMOsis scl3 and CRL-7566 cells were identified as ER and PR positive as with EMOsis cc/TERT cells and CRL-4003 cells. (B) We examined that the mRNA expression of decorin of HMOsis scl2, HMOsis scl3 and CRL-7566 cells with or without dienogest treatment. The HMOsis scl2, HMOsis scl3 and CRL-7566 cells treated with dienogest showed significantly higher expression of decorin mRNA. (PDF 63 KB)
