# Supplementary figures and images for: Decorin induced by progesterone plays a crucial role in suppressing endometriosis
Source: J Endocrinol. 2014 Nov;223(2):203–16. doi: 10.1530/JOE-14-0393 (PMC4198121; doi:10.1530/JOE-14-0393)

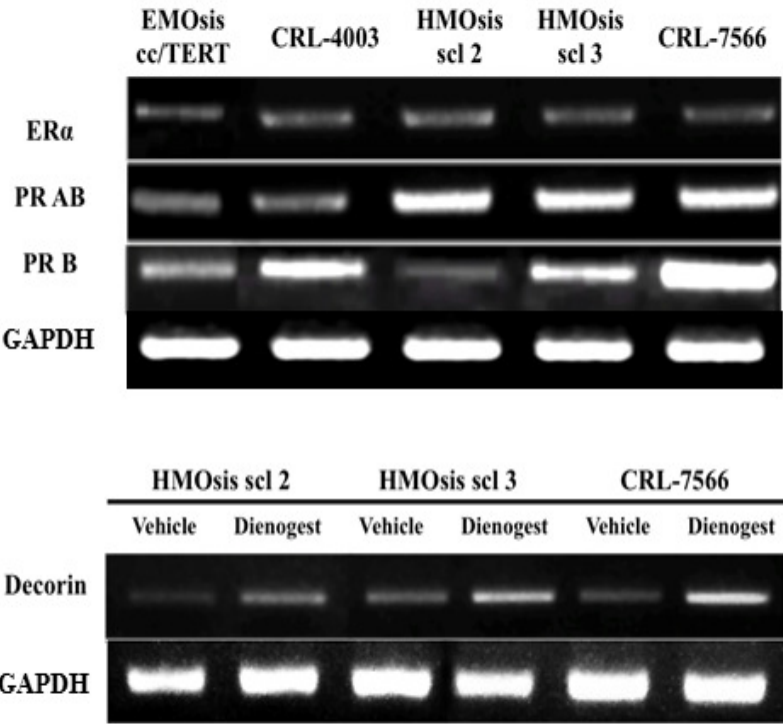

Supplement: Supplementary Figure [file supp_JOE-14-0393_Supplementary_figure_1.pdf]
